# Do Primocolonizing Bacteria Enable Bacteroides thetaiotaomicron Intestinal Colonization Independently of the Capacity To Consume Oxygen?

**DOI:** 10.1128/mSphere.00232-19

**Published:** 2021-05-05

**Authors:** David Halpern, Claire Morvan, Aurélie Derré-Bobillot, Thierry Meylheuc, Mélanie Guillemet, Sylvie Rabot, Alexandra Gruss

**Affiliations:** a Université Paris Saclay, INRAE, Micalis Institute, MicrobAdapt, Jouy en Josas, France; b Laboratoire Pathogenèses des Bactéries Anaérobies, Institut Pasteur, UMR CNRS 2001, Université de Paris, Paris, France; c Université Paris Saclay, INRAE, Micalis Institute, MIMA2, Jouy en Josas, France; d Université Paris Saclay, INRAE, Micalis Institute, Anaxem, Jouy en Josas, France; University of California, Davis

**Keywords:** *Bacteroides*, *Clostridium scindens*, *Escherichia coli*, germfree mice, intestine, oxygen, primocolonization

## Abstract

Aerobic bacteria are frequent primocolonizers of the human naive intestine. Their generally accepted role is to eliminate oxygen, which would allow colonization by anaerobes that subsequently dominate bacterial gut populations. In this hypothesis-based study, we revisited this dogma experimentally in a germfree mouse model as a mimic of the germfree newborn. We varied conditions leading to the establishment of the dominant intestinal anaerobe Bacteroides thetaiotaomicron. Two variables were introduced: *Bacteroides* inoculum size and preestablishment by bacteria capable or not of consuming oxygen. High *Bacteroides* inoculum size enabled its primocolonization. At low inocula, we show that bacterial preestablishment was decisive for subsequent *Bacteroides* colonization. However, even non-oxygen-respiring bacteria, a *hemA* Escherichia coli mutant and the intestinal obligate anaerobe Clostridium scindens, facilitated *Bacteroides* establishment. These findings, which are supported by recent reports, revise the long-held assumption that oxygen scavenging is the main role for aerobic primocolonizing bacteria. Instead, we suggest that better survival of aerobic bacteria *ex vivo* during vectorization between hosts could be a reason for their frequent primocolonization.

## Abstract (precis)

Aerobic bacteria are frequent primocolonizers of the human naive intestine. Their generally accepted role is to eliminate oxygen, which would allow colonization by anaerobes that subsequently dominate bacterial gut populations.

## OPINION/HYPOTHESIS

Initial microbial colonization of the naive intestine may have lasting consequences on the host (1, 2), yet the factors that influence this crucial step are mainly unknown (3). The temporal sequence of microbial establishment varies greatly among individual human newborns (4–6). The concentration and composition of the microbial bolus encountered by neonates and the uniqueness of each individual are likely crucial to the colonization of the naive intestine, making the identification of factors governing colonization a major challenge.

*Bacteroides* species are dominant heme auxotrophs and obligate anaerobes of human and animal intestinal microbiota (7–10), which coexist in symbiosis with the healthy host. These bacteria are proposed to contribute to host well-being, e.g., by (i) providing membrane-permeable nutrients such as short-chain fatty acids, (ii) occupying the intestinal mucosal space and thus preventing access to pathogens (this role relies on a large repertoire of *Bacteroides* enzymes that catabolize complex sugars lining the intestinal mucosal wall), and (iii) producing antimicrobial molecules that may limit the outgrowth of bacterial competitors, including pathogens (1, 11–13).

Oxygen depletion in the intestine by precolonizing bacteria is considered the *sine qua non* for Bacteroides thetaiotaomicron colonization. Aerobic bacteria such as Escherichia coli, which are often among the primocolonizers, are proposed to be responsible for consuming toxic oxygen, thus enabling subsequent B. thetaiotaomicron establishment (4, 14). However, to our knowledge, this dogma remains unproven. Moreover, microbial footprints of neonate feces indicate that aerobes are not systematically the first to colonize the intestines (5). In this work, we therefore revisit this hypothesis by giving evidence in a germfree mouse model that primocolonizing bacteria promote B. thetaiotaomicron establishment regardless of their capacity to consume oxygen.

### B. thetaiotaomicron primocolonization of the mouse intestine is inoculum dependent.

**FIG 1:**
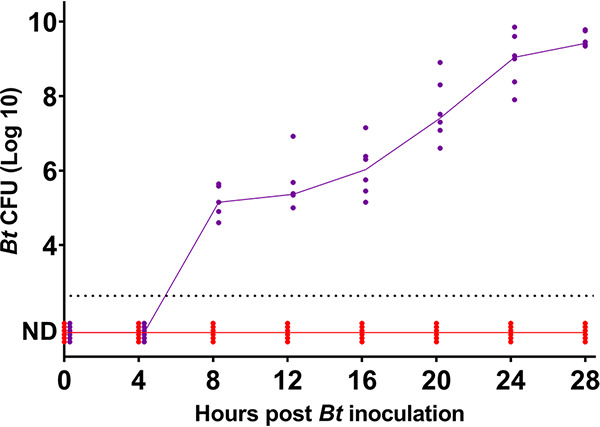
B. thetaiotaomicron (*Bt*) implantation in the intestine as a function of inoculum size. B. thetaiotaomicron was administered orally to germfree BALB/c mice at two concentrations by gastric probe. Fecal samples were taken at the time of implantation and every 4 h for 28 h. Oral administration was with 10^3^ CFU B. thetaiotaomicron (red) or 10^4^ CFU B. thetaiotaomicron (purple). Individual values are shown for each time point; the intersection of lines with values indicates the median CFU per gram in fecal samples of mice for each cohort. The detection threshold was 5 × 10^2^ CFU/g feces. ND, not detected. Data for mice where no CFU were detected are expanded at the baseline to distinguish the number of mice tested.

Most colonization studies involving B. thetaiotaomicron use 10^6^ to 10^8^ CFU for implantation (15). We reasoned that under natural conditions, B. thetaiotaomicron concentrations that reach the intestines might be far lower. Even if higher concentrations are ingested at childbirth, contact with gastric products during passage through the intestine could decrease microbial survival (16, 17). All methodologies are described in Text S1 in the supplemental material. Accordingly, 10^3^ and 10^4^ CFU of B. thetaiotaomicron, determined by first establishing the correlation with optical density at 600 nm (OD_600_) readings, were orally administered at time zero (*T*_0_) to two mouse cohorts (*n* = 6). B. thetaiotaomicron colonization was assessed by CFU determinations in feces, sampled at 4-h intervals for 28 h, starting at *T*_0_. The capacity to colonize was found to be inoculum dependent (Fig. 1). Administration of 10^4^ CFU led to colonization at 8 h postinoculation (p.i.), whereas the 10-fold-lower concentration did not promote B. thetaiotaomicron establishment even at 28 h p.i. These findings suggest that inoculum size is a contributing factor for B. thetaiotaomicron primocolonization.

10.1128/mSphere.00232-19.1 TEXT S1 Word file describing the materials and methods used for experimentation: strains, constructions, growth conditions, oxygen consumption measurements, colonization of germfree mice, determination of colonization efficiency, and microscopy. Download Text S1, PDF file, 0.2 MB. Copyright © 2021 Halpern et al. 2021 Halpern et al. https://creativecommons.org/licenses/by/4.0/ This content is distributed under the terms of the Creative Commons Attribution 4.0 International license.

### E. coli enables B. thetaiotaomicron colonization in a germfree mouse intestinal model.

**FIG 2:**
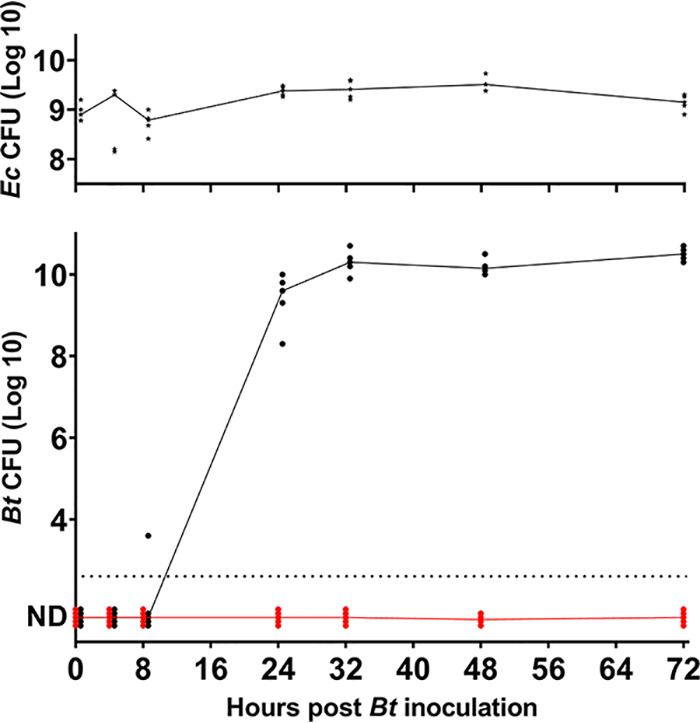
E. coli facilitates B. thetaiotaomicron (*Bt*) establishment in germfree animals. E. coli MG1655 (WT) was established in germfree BALB/c mice by oral administration. Sixteen hours later (*T*_0_), 2 × 10^3^ CFU of B. thetaiotaomicron were administered to the group precolonized by E. coli and to a second naive group. All mouse groups received the B. thetaiotaomicron doses at the same time and from the same bacterial preparation. Fecal samples were taken at the indicated times over a 72-h period for CFU determinations. Dilutions were spotted on *Bacteroides* bile esculin agar with amikacin (BBE) medium incubated anaerobically for B. thetaiotaomicron and on LB medium incubated aerobically for E. coli. (Top) E. coli (*Ec*) CFU per gram; (bottom) B. thetaiotaomicron CFU per gram of feces. Red, B. thetaiotaomicron administered alone; black, B. thetaiotaomicron administered after E. coli precolonization. Individual values are shown for each time point; the intersection of lines with values indicates the median CFU per gram in fecal samples of the mice for each cohort. The detection threshold was 5 × 10^2^ CFU/g feces. ND, not detected. Data for mice where no CFU were detected are expanded at the baseline to distinguish the number of mice tested.

Although Escherichia coli is a minor constituent of the adult microbiota, it is frequently among the first species to transiently dominate the naive newborn intestinal microbiota (4, 5, 18). E. coli is unique among the major intestinal bacteria to be fully equipped for aerobic respiration and to thereby eliminate oxygen (19, 20). We examined the capacity of the “low” B. thetaiotaomicron inoculum (10^3^ CFU) to colonize intestines of mice that were preimplanted (16 h prior to the B. thetaiotaomicron inoculum [*T*_−16_]) or not with E. coli strain MG1655 (10^8^ CFU) (Fig. 2). As mentioned above, no B. thetaiotaomicron bacteria were detected in feces of monocolonized mice when sampled up to 72 h p.i. In marked contrast, mice preimplanted with E. coli were colonized by B. thetaiotaomicron at 10^9^ to 10^10^ CFU calculated per g of feces at 24 h p.i. This range is comparable to the CFU reported after mouse colonization with a high B. thetaiotaomicron inoculum (2 × 10^10^ CFU) (15).

**FIG 3:**
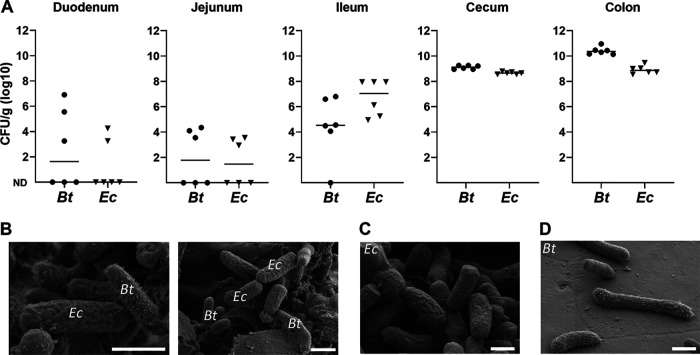
E. coli and B. thetaiotaomicron (*Bt*) colocalize in the mouse intestinal tract. (A) Bacterial loads in intestinal compartments. Intestinal samples were recovered from E. coli WT (*Ec*)- and B. thetaiotaomicron-cocolonized mice used in the experiment shown in Fig. 2, 72 h after the start of experiments. Intestinal contents were recovered from the five indicated locations of dissected mice, and CFU were determined. Bars represent the median values of CFU obtained from individual samples. ND, below the detection level. (B) Visualization by field emission scanning electron microscopy of feces from cocolonized mice. E. coli and B. thetaiotaomicron are identified by their distinct morphologies. Small particles may correspond to food particles or shed mucus. (C and D) Purified cultures were used for identification. White bars, 1 μM.

The marked impact of E. coli on B. thetaiotaomicron colonization at low inocula (Fig. 2) might suggest the proximity of the two species in the gut. Bacterial loads in cocolonized mice were determined from the different intestinal compartments (Fig. 3A). For each given compartment, E. coli and B. thetaiotaomicron showed comparable CFU, ranging from about 10^2^ to 10^3^ CFU/g in the duodenum and jejunum to 10^9^ to 10^11^ CFU/g in the cecum and colon. Scanning microscopy of feces of cocolonized mice (Fig. 3B) revealed two discrete bacterial forms, which were distinguishable as E. coli and B. thetaiotaomicron, as identified in monocultures (Fig. 3C and D). B. thetaiotaomicron and E. coli contact and metabolic exchanges were suggested and shown to occur in dysbiosis and infection (21, 22). The proximity of these bacteria as observed here suggests that similar exchanges are possible in the healthy host in early stages of colonization.

### E. coli facilitates B. thetaiotaomicron colonization independently of a role as an oxygen scavenger.

**FIG 4:**
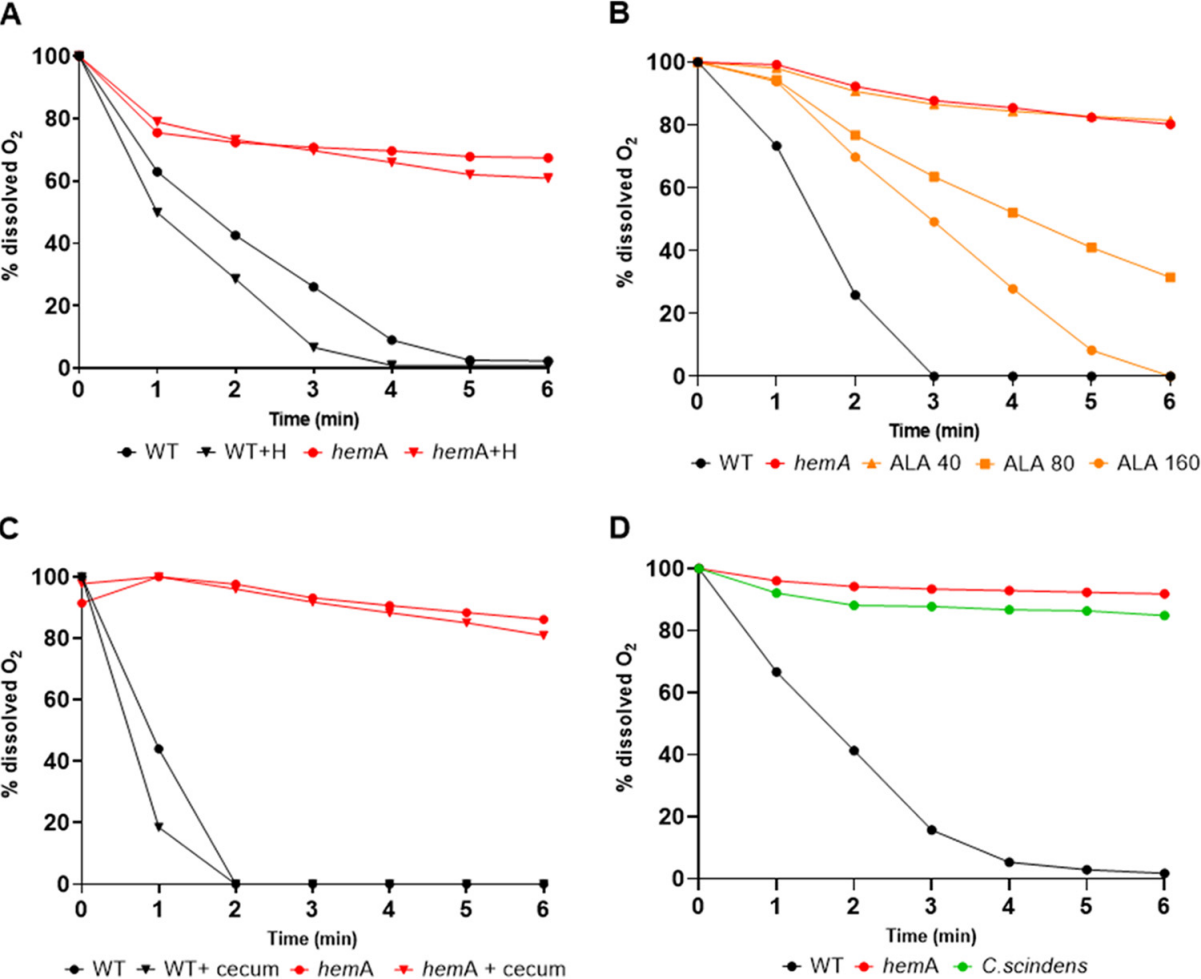
Bacterial oxygen consumption. (A) E. coli MG1655 and *hemA* strains were grown in LB supplemented or not with 5 μM heme (H). (B) E. coli WT and *hemA* strains were grown in LB. The *hemA* strain was also grown in LB supplemented with the indicated concentrations of δ-aminolevulinic acid (ALA) (micromolar). (C) WT and *hemA* E. coli strains were grown in LB or in 90% murine cecum containing 10% of a 10×-concentrated LB medium. (D) *C. scindens* and E. coli WT and *hemA* control strains were compared for their capacity to consume oxygen. See Text S1 in the supplemental material for protocols. Dissolved oxygen (milligrams per liter) is normalized to 100% for all samples at *T*_0_.

B. thetaiotaomicron growth is inhibited by oxygen, which led to the simple and generally accepted hypothesis that respirative aerobic bacteria consume intestinal oxygen, thus facilitating the subsequent implantation of anaerobes such as B. thetaiotaomicron (14). We tested this hypothesis by assessing B. thetaiotaomicron establishment in germfree mice precolonized by an E. coli strain that does not consume oxygen, compared to a wild-type (WT) E. coli strain. We chose a *hemA* mutant, which does not synthesize heme and thus cannot carry out aerobic respiration, the main pathway for oxygen reduction to water (19). Unlike other respiration-related genes, which are mostly redundant in E. coli, the *hemA* mutation disables respiration and oxygen-consuming functions (19). It also disables anaerobic respiration by nitrate, which is reportedly used in the gut upon inflammation (23). This choice allowed us to inactivate a single rather than multiple genes without compromising fermentation growth. We first validated the differences in oxygen consumption of the MG1655 WT and *hemA* mutant strains. As expected, only the WT strain consumed oxygen (Fig. 4). It was possible that intestinal heme (24) or δ-aminolevulinic acid (ALA) (the HemA product) (25) could alter the capacity of the *hemA* strain to consume oxygen. However, heme addition did not affect *hemA* mutant oxygen consumption, which is consistent with observations that MG1655 does not assimilate exogenous heme (26, 27) (Fig. 4A). In contrast, while ALA has not, to our knowledge, been reported in intestinal contents, it was detected in blood plasma at trace levels (<0.1 μM in healthy humans [28]) and in urine (up to ∼20 μM in healthy individuals [29]). The MG1655 *hemA* mutant consumed oxygen in the presence of 80 μM to 160 μM ALA but not at 40 μM ALA (Fig. 4B). To determine whether intestinal contents might stimulate *hemA* oxygen consumption, WT and *hemA* strains were grown in a pooled murine cecal sample, and oxygen consumption was measured (Fig. 4C). Cecum addition had no effect on WT strain oxygen consumption and had no stimulatory effect on oxygen consumption by the *hemA* strain. We therefore considered that *hemA* would not consume oxygen during gut passage.

**FIG 5:**
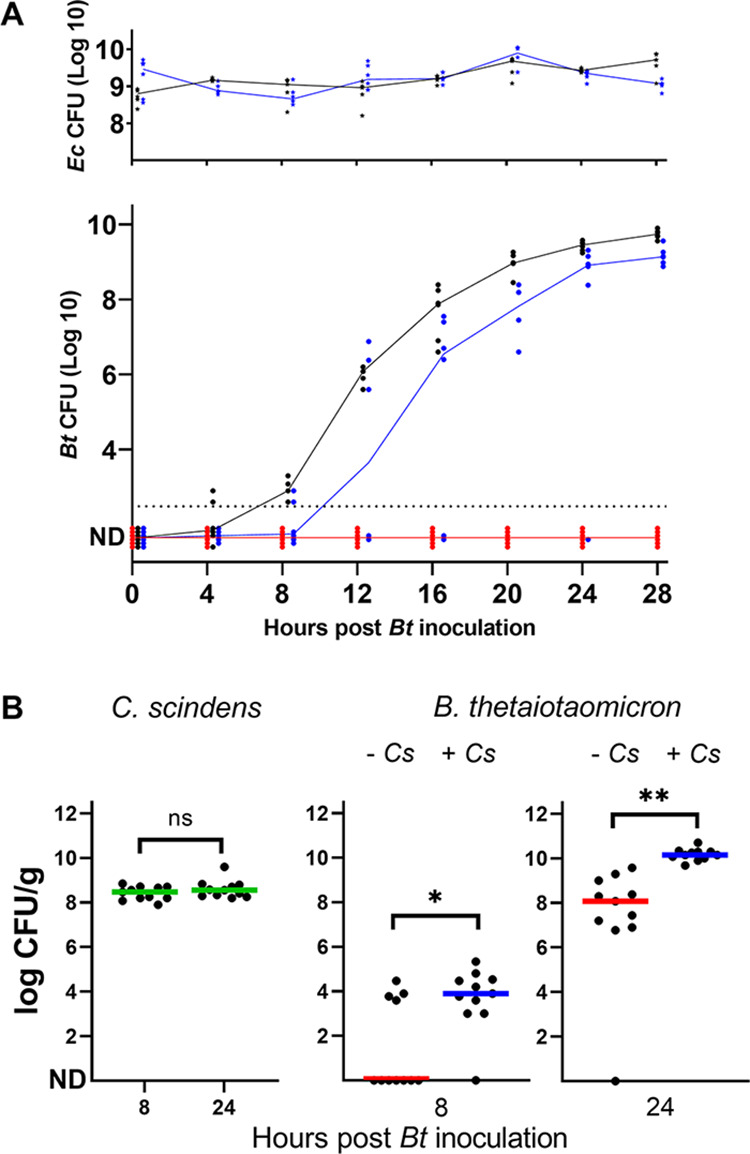
Precolonizing aerobic and anaerobic bacteria facilitate B. thetaiotaomicron (*Bt*) establishment in germfree mice. (A) E. coli WT and *hemA* mutant strains (10^8^ CFU) were each orally administered to germfree BALB/c mice. Sixteen hours later (*T*_0_), 2 × 10^3^ CFU of B. thetaiotaomicron were administered to the two groups of animals precolonized with E. coli and to a group of naive mice. All mouse groups received the B. thetaiotaomicron doses at the same time and from the same bacterial preparation. Fecal samples were taken at 4-h intervals for 28 h for CFU determinations. E. coli *hemA* CFU were determined on LB plates containing δ-aminolevulinic acid (200 μM) incubated aerobically. (Top) E. coli (*Ec*) (CFU per gram feces); (bottom) B. thetaiotaomicron (CFU per gram of feces). Red, mice colonized with B. thetaiotaomicron alone; black, mice colonized with E. coli WT and then B. thetaiotaomicron; blue, mice colonized with E. coli *hemA* and then B. thetaiotaomicron. Individual CFU values are shown for each time point; the intersecting line represents the median of CFU for each cohort. The detection threshold was 5 × 10^2^ CFU/g feces. ND, not detected. Data for mice where no CFU were detected are expanded at the baseline to distinguish the number of mice tested. (B) Anaerobic *C. scindens* (*Cs*) was administered as described above for panel A for the administration of E. coli to germfree animals, while a second group of mice received no *C. scindens* as a control. Sixteen hours later, both groups of mice received 10^3^ CFU B. thetaiotaomicron by oral administration. CFU determinations were performed at 8 h and 24 h p.i. (Left) *C. scindens* CFU at 8 h and 24 h in fecal samples of mice precolonized with this bacterium. (Right) B. thetaiotaomicron CFU at 8 h and 24 h in feces samples of mice with (+ *Cs*) or without (− *Cs*) *C. scindens* precolonization. Results of two independent experiments were pooled. Bars indicate median CFU per gram for the mice in each group. The detection threshold was 5 × 10^2^ CFU/g feces. ND, below the detection level. *, *P* = 0.5; **, *P* = 0.05; ns, not significant.

The capacity of the *hemA* mutant to enable B. thetaiotaomicron colonization was tested in the germfree mouse model as described above. Mice were precolonized (*T*_−16_) with either the MG1655 WT or the *hemA* strain. A third group of germfree mice was not precolonized. At *T*_0_, all groups were administered 2 × 10^3^ CFU of B. thetaiotaomicron. Fecal samples were collected at 4-h intervals over a 28-h period for E. coli and B. thetaiotaomicron CFU determinations (Fig. 5A). As described above, B. thetaiotaomicron only colonized mice that were precolonized with E. coli. In mice precolonized with the *hemA* mutant, compared to the WT E. coli strain, B. thetaiotaomicron establishment was delayed by about 4 h. The *hemA* strain phenotypes (kanamycin resistance and no growth on aerobically incubated solid medium) were confirmed in bacteria recovered from feces at the 28-h time point, indicating that the strain did not revert to the WT in the gut. The E. coli *hemA* strain thus had nearly the same stimulatory effect on B. thetaiotaomicron establishment as did WT E. coli. These findings suggest a marginal, if any, role for E. coli as an oxygen scavenger in promoting B. thetaiotaomicron establishment. These *in vivo* findings argue against the currently accepted hypothesis that respiratory aerobic bacteria eliminate toxic oxygen from the intestine to facilitate *Bacteroides* establishment.

The role of accessory bacteria in enabling B. thetaiotaomicron establishment was then investigated using Clostridium scindens, an obligate anaerobe and common isolate of the healthy human intestine (30), in place of E. coli as a primocolonizer. As expected, the tested C. scindens strain ATCC 35704 did not consume oxygen (Fig. 4D). The capacity of B. thetaiotaomicron to colonize mouse intestines was tested as described above, in the absence or presence of *C. scindens*. In these experiments, which were performed twice independently, B. thetaiotaomicron CFU appeared even in the absence of precolonizing bacteria. This observed shift might be related to a change in germfree BALB/c mouse suppliers and/or to subtle changes in animal housing conditions that occur over time (e.g., water or food supply). Nevertheless, precolonization with *C. scindens* significantly improved B. thetaiotaomicron establishment (Fig. 5B). Altogether, these findings rule out species specificity and demonstrate that oxygen consumption by aerobic bacteria is not a *sine qua non* for B. thetaiotaomicron establishment.

### Limitations of the primocolonization germfree model.

To our knowledge, this is the first description of a germfree model that tests intestinal primocolonization with low bacterial doses. In developing this approach, we confronted two notable technical issues. The first concerns the use of low inocula: while great care was taken to ensure reproducible conditions, the use of low inocula increases the risk of variation during inoculation and amplifies differences between individuals within a cohort. The second concerns the handling of anaerobic bacteria, which are oxygen sensitive. After anaerobic growth, B. thetaiotaomicron bacteria are briefly exposed to oxygen during inoculum preparation for oral administration. These steps need careful coordination to ensure repeatability and minimize the period of oxygen exposure. The combination of these limitations was considered when choosing the minimal B. thetaiotaomicron colonization dose (1 × 10^3^ to 2 × 10^3^ CFU per mouse) and by simultaneously administering doses from a single bacterial stock. We recommend that these technical steps be carefully prepared and timed in experimentations involving low-dose bacterial administrations, particularly when dealing with anaerobic bacteria.

### Anaerobic bacteria may encode functions involved in oxygen management.

Properties of B. thetaiotaomicron itself might suggest why bacterially mediated oxygen removal is not needed for its establishment: (i) B. thetaiotaomicron encodes an aerobic respiration system involving quinol oxidase, which allows it to withstand nanomolar concentrations of oxygen (shown for the closely related species Bacteroides fragilis [31]); (ii) B. thetaiotaomicron and B. fragilis encode a catalase and other peroxide-scavenging enzymes, which may eliminate toxic oxygen radicals (32, 33); and (iii) frequently arising mutations in *oxe* (BF638R_0963), a B. fragilis flavoprotein, reportedly led to greater oxygen resistance and are common in clinical isolates (B. thetaiotaomicron carries an *oxe* homolog [BT_4126] sharing 92% identity [34]). Moreover, B. thetaiotaomicron colonizes germfree rats when the oxidoreduction potential is high, in keeping with its tolerance to an oxidative environment (15). Importantly, *C. scindens* is itself anaerobic and was directly established in the mouse intestine albeit at a high inoculum (Fig. 5B), further supporting the proposal that oxygen removal is not the main role of primocolonizing bacteria.

### Further studies point to alternative roles of primocolonizing bacteria, without direct oxygen consumption.

The above-described results revise the accepted main role of primocolonizing bacteria and raise questions on their roles in enabling B. thetaiotaomicron establishment without involving respiratory oxygen consumption (Fig. 1). This function is not E. coli specific and can be fulfilled by an anaerobic bacterium, as shown here with *C. scindens*. Colonization is associated with rapid changes in intestinal volume and cell histology (35, 36), some within hours of colonization, as well as changes in mucus glycan composition and the production of metabolites (11, 24, 36–38). Evidence for an indirect modulation of intestinal oxygen homeostasis by bacteria is suggested from recent studies. Interestingly, bacterial pathogens, but also the normal microbiota, may trigger an anoxic response, depleting oxygen in their surrounding tissues. The bacterial metabolite butyrate, which is produced by anaerobic bacteria, was proposed to stimulate oxygen elimination via β-oxidation in host cells (see reference 39 and references therein; 40, 41). More generally, lipid β-oxidation triggered by the microbiota was suggested as a means of removing oxygen (42), further supporting an alternative role for primocolonizing bacteria in modulating intestinal oxygen. Interestingly, previous studies also give evidence that no notable differences in oxygen status exist between germfree and conventional intestines, further questioning the need for oxygen consumption by aerobic bacteria (42, 43). These and our conclusions are also consistent with an exhaustive study of primocolonizing bacteria in human neonates, where in some babies, the dominant primocolonizing bacteria were members of *Bacteroidetes* genera (5). In a simpler hypothesis that reconciles our and previous findings, we suggest that aerobic bacteria have a better chance of survival *ex vivo*, during transmission between donor and recipient. This is consistent with (i) recent studies indicating that intestinal E. coli bacteria develop essentially by anaerobic growth (44) and (ii) observations of a greater abundance of aerobic bacteria in babies born by Caesarean than in babies born by vaginal delivery (45).

### Importance of oxygen consumption in infection conditions?

While our findings rule out the need for aerobic respiring bacteria during primocolonization, this property may be important in other situations. For example, intestinal dysbiosis due to infection, postantibiotic treatment, or inflammation might lead to high E. coli populations (46–48). The proximity of E. coli to B. thetaiotaomicron in the dysbiotic host could increase the availability of metabolites (e.g., bacterial growth-promoting heme and quinones [24, 49]) and may also protect anaerobes in the stressed host by respiring oxygen. Oxygen elimination by aerobic bacteria might thus be relevant to *Bacteroides* survival during polymicrobial intra-abdominal infection (22, 50).
