# Supplementary material for: Do Primocolonizing Bacteria Enable Bacteroides thetaiotaomicron Intestinal Colonization Independently of the Capacity To Consume Oxygen?
Source: mSphere. 2021 May 5;6(3):e00232-19. doi: 10.1128/mSphere.00232-19 (PMC8103986; doi:10.1128/mSphere.00232-19)
Supplement: TEXT S1 [file mSphere.00232-19-s0001.pdf]

Supplementary Information

Do primocolonizing bacteria enable *Bacteroides thetaiotaomicron* intestinal colonization independently of the capacity to consume oxygen?

David Halpern[1], Claire Morvan[2], Aurélie Derré-Bobillot[1], Thierry Meylheuc[3], Mélanie Guillemet[1], Sylvie Rabot[4], and Alexandra Gruss[1†]

[1] Université Paris Saclay, INRAE, Micalis Institute, MicrobAdapt, Jouy en Josas, France [2] Laboratoire Pathogenèses des Bactéries Anaérobies, Institut Pasteur, UMR CNRS 2001, Université de Paris, Paris, France [3] Université Paris Saclay, INRAE, Micalis Institute, MIMA2, Jouy en Josas, France, [4] Université Paris Saclay, INRAE, Micalis Institute, Anaxem, Jouy en Josas, France

Material and Methods

Strains, constructions, and growth conditions

B. thetaiotaomicron strain VPI-5482 was originally isolated from the feces of a healthy human adult [1]. *B. thetaiotaomicron* cultures were routinely started from 50 µl frozen aliquots prepared as follows: An overnight culture was prepared in M17-glu (0.5 % glucose) containing heme (10 µM) and cysteine (4 mM); the culture was washed and resuspended in fresh M17 medium containing 15 % glycerol and then aliquoted anaerobically and stored at -80°C. For all experiments, *B. thetaiotaomicron* cultures were prepared as follows: 10 µl of a frozen aliquot was thawed and resuspended in 10 ml M17-glu without heme and grown overnight at 37°C in an anaerobic chamber (Sheldon Manufacturing Inc., Cornelius Ore). Cultures were then used as below for each experiment.

E. coli strain MG1655 is a K-12 laboratory strain derived from a stool culture (http://www.genome.wisc.edu/resources/strains.htm). The MG1655 *hemA* mutant was constructed by P1 phage transduction from the *E. coli* C600 *hemA::km* (kanamycin resistant gene insertion) strain (Wandersman lab collection; Institut Pasteur, France; [2]). *E. coli* MG1655 wild type (WT) and *hemA* strains were routinely grown in LB medium supplemented with δ-aminolevulinic acid (ALA; 200 µM). Kanamycin (50 µg/ml) was added to verify the *hemA::km* insertion.

Clostridium scindens is an obligate anaerobe and a major bile acid 7α-dehydroxylating bacterium that is a predominant bacterial constituent of healthy human fecal microbiota [3]. *C. scindens* strain ATCC 35704 was grown in BHI solid and liquid medium at 37°C in an anaerobic chamber.

Oxygen consumption measurements

To test effects of ALA and cecum on *E. coli* WT and *hemA* oxygen consumption, strains were first streaked on LB-ALA medium. Single colonies were used to prepare overnight static cultures in M17-glu. Cultures were then diluted 1/100 in M17-glu medium containing 0, 40, 80, or 160 µM ALA. For experiments where cecum was added, cecal content was removed under sterile dissection conditions from 20 germ-free mice, pooled, and frozen at -20°C prior to use. A slurry was prepared using 90 % cecum and 10% of a 10X-concentrated LB medium plus 0.5 % glucose; the slurry was centrifuged at

10,000 rpm and the supernatant was used as *E. coli* culture medium. Cultures were grown for 4 - 6 hours in an anaerobic jar at 37°C. Cells were then centrifuged and resuspended in PBS buffer at 4°C to obtain 1 ml bacterial suspensions at OD_{600} = 1.0. Oxygen consumption was monitored over 6 minutes at 37°C with aeration using a Clark-type oxygen electrode (Liquid-Phase Oxygen electrode unit DW1, Hansatech instruments, UK) as described [4], except that readings were done upon addition of 2.5 % glucose. Oxygen consumption by *E. coli* WT and *hemA* strains in the absence or presence of heme (5 µM), or by *C. scindens*, was measured as follows. Bacteria were first streaked on LB-ALA for *E. coli* WT and *hemA* as above; *C. scindens* was grown in BHI and incubated for 48 h at 37°C in an anaerobic chamber. Single colonies were used to prepare overnight anaerobic liquid cultures at 37°C in M17-glu (for *E. coli*) or in BHI (*C. scindens*). Cultures were diluted (1/100) and grown to OD_{600} between 0.5 and 0.7. Cultures were then removed from the anaerobic chamber and vigorously shaken to saturate the medium with oxygen. Note that here, we monitored enzymatic capacity to eliminate oxygen; survival of *C. scindens* was not expected with this treatment, and was not tested. Oxygen consumption was followed using a HQ40D unit with an LDO probe (HACH Company, Loveland, Co). The LDO probe was immersed in the bacterial culture maintained at 37°C and the decrease in the amount of dissolved oxygen was followed for 6 minutes. For each sample, results were normalized to 100 % as greatest oxygen content.

Colonization of germ-free mice

Germ-free BALB/c mice were bred at the Anaxem animal facility (Micalis, INRAE, Jouy en Josas, France; license number: B 78-322-6), and maintained in gnotobiotic isolators under prescribed conditions. The BALB/c mice were purchased from Charles River Laboratories (L'Arbresle, France) and rendered germ-free at Anaxem; BALB/c mice used for experiments with *C. scindens* were purchased germ-free from Taconic Biosciences (Rensselaer, NY). The Anaxem platform checks the sterile status of its mice, using optical microscopic examination of 100-fold dilutions of freshly voided feces and bacteriological culturing of the feces in various conditions (several non-selective liquid and agar media incubated at different temperatures in aerobic and anaerobic conditions). For each cage environment, sterility is

confirmed using a "sentinel" mouse, followed by the fecal tests described above. Thus, mouse and isolator status are confirmed prior to all tests. Experiments were carried out in accordance with the European Guidelines for the Care and Use of Laboratory Animals and approved by the Ethics Committee of the INRAE Research Center at Jouy-en-Josas (approval references: APAFIS#4595-2016010815599428 v4 and APAFIS#23311-2016010815599428 v9). Animals were fed on standard chow (irradiated at 45 kGy; R03-40, SAFE, France http://www.safe-diets.com/fr/accueil/). Bacteria were administered orally in 100 µl volumes using flexible needles (ECIMED, Boissy-St-Leger, France). Seven to ten week old male mice were used in groups of five or six per experimental condition in all experiments.

Sealed tubes containing bacterial samples and required materials to be transferred to the isolator are introduced through the isolator airlock, sprayed with a freshly prepared solution of 4 % peracetic acid and incubated 30 min prior to their introduction in the isolator. This standardized procedure prevents risks of contamination.

E. coli and *C. scindens* cultures used for pre-colonization were prepared respectively in M17-glu at 37°C in static conditions, and in BHI medium in an anaerobic chamber. Overnight cultures were diluted 1:100 and grown to mid-exponential phase. Cultures were centrifuged and resuspended to obtain 1-2 x 10^8 colony forming units (CFU) in 100 µl 0.9 % NaCl for oral administration. *B. thetaiotaomicron* VPI-5482 cultures used for inoculations were prepared from overnight cultures, centrifuged, and resuspended according to OD_{600} to the required concentration in 0.9 % NaCl in a 100 µl volume.

At 16 h after pre-colonization, pre-colonized and control mice were inoculated with *B. thetaiotaomicron* (1-2x10^3 or 2x10^4 CFU); this time is called T0. To determine CFU, we first established the correlation between *B. thetaiotaomicron* CFUs and OD_{600} by plating experiments (OD_{600} = 1 corresponds to 2± 1.8 x 10^8 CFU, N=5). Due to technical limits of timing for live experiments in germ-free isolators, we then relied on OD_{600} readings to calculate CFUs for inocula in all experiments. We note that unavoidable bacterial exposure to O_2 occurs just prior to administration. Two precautions

were taken: first, all mouse groups were administered *B. thetaiotaomicron* simultaneously (one technician per animal group), and second, all experiments included the reference controls. During experiments, feces were collected just before *E. coli* colonization, at T0, and at specified time intervals. In dissection experiments on co-colonized animals, animals were sacrificed 72 hours after the start of experiments. Intestinal contents were recovered for CFU determinations and microscopy.

Determination of colonization efficiency

Directly after feces sampling, and for recovery of intestinal contents, samples were transferred to an anaerobic chamber and resuspended as a 1:10 dilution in 0.9 % NaCl. Ten-fold dilutions were spotted (5 µl per spot) for CFU determinations. *B. thetaiotaomicron* CFU were determined on selective BBE agar plates (Bacteroides Bile Esculine agar with Amikacin; Becton Dickinson) incubated 48 h at 37°C in an anaerobic chamber. *E. coli* WT dilutions were spotted on LB solid medium. *E. coli hemA* dilutions were spotted on LB containing kanamycin, plus 200 µM ALA, which restores heme synthesis and allows aerobic growth. Plates were incubated aerobically at 37°C for 24 h. Maintenance of the *hemA* phenotype was confirmed at the last feces sampling point by comparing CFU plating on LB medium without and with ALA. *C. scindens* CFU determinations were done anaerobically on BHI solid medium. Mann and Whitney unpaired 2-tailed tests were used to analyze statistical relevance of all data analyzed from mouse studies (GraphPad Prism; Graphpad Software Inc., USA).

Microscopy

Field emission scanning electron microscopy was performed to visualize *E. coli* and *B. thetaiotaomicron* in mouse feces. Feces were collected at T48 h, and prepared for microscopy by chemical dehydration: samples were immediately immersed in a fixative solution (2.5 % glutaraldehyde in 0.2 M sodium cacodylate buffer, pH 7.4), deposited on sterile cover-glass disks (Marienfeld, VWR, France) and stored 1 h at room temperature, then overnight at 4°C. The fixative was removed, and samples were rinsed three times for 10 min in sodium cacodylate buffer. Samples underwent progressive dehydration by soaking in a graded series of ethanol (50 % to 100 %) baths before drying under CO_2. Samples were mounted on aluminum blocks (10 millimeter diameter) with conductive silver paint and sputter coated

with gold-palladium (Polaron SC7640; Elexience, Verrières-le-Buisson, France) for 200 s at 10 mA. Samples were viewed as secondary electron images (2 kV) with a Hitachi S4500 instrument (Elexience, Verrières-le-Buisson, France). Analyses were performed at the Microscopy and Imaging Platform MIMA2 (Micalis, INRAE, Jouy en Josas, France).

References

1. Xu J, Bjursell MK, Himrod J, Deng S, Carmichael LK, Chiang HC, et al. A genomic view of the human-*Bacteroides thetaiotaomicron* symbiosis. Science. 2003;299(5615):2074-6. Epub 2003/03/29. doi: 10.1126/science.1080029. PubMed PMID: 12663928.
2. Letoffe S, Debarbieux L, Izadi N, Delepelaire P, Wandersman C. Ligand delivery by haem carrier proteins: the binding of *Serratia marcescens* haemophore to its outer membrane receptor is mediated by two distinct peptide regions. Mol Microbiol. 2003;50(1):77-88. Epub 2003/09/26. doi: 3686 [pii]. PubMed PMID: 14507365.
3. Morris G, Winter J, Cato E, Ritchie A, Bokkenheuser VD. *Clostridium scindens* sp*. nov.*, a Human Intestinal Bacterium with Desmolytic Activity on Corticoids. Intl J Systematic Bacteriol. 1985;35:478-81.
4. Yamamoto Y, Poyart C, Trieu-Cuot P, Lamberet G, Gruss A, Gaudu P. Respiration metabolism of Group B Streptococcus is activated by environmental haem and quinone and contributes to virulence. Mol Microbiol. 2005;56(2):525-34. PubMed PMID: 15813741.
